# Facilitators of and barriers to collaboration between universities and the food industry in nutrition research: a qualitative study

**DOI:** 10.29219/fnr.v65.7874

**Published:** 2021-10-27

**Authors:** Lisa Garnweidner-Holme, Helle Skoglund Lieberg, Harald Irgens-Jensen, Vibeke H. Telle-Hansen

**Affiliations:** 1 Department of Nursing and Health Promotion, Faculty of Health Sciences, Oslo Metropolitan University, Oslo, Norway; 2 Department of Private Law, Faculty of Law, University of Oslo, Oslo, Norway

**Keywords:** research collaboration, nutrition science, food industry, qualitative research

## Abstract

**Background:**

Unhealthy food is one of the main risk factors for non-communicable diseases. Improved knowledge about healthy and sustainable food products requires nutrition research in collaboration between universities and the food industry.

**Objective:**

To investigate the facilitators of and barriers to university–industry collaborations in nutrition research.

**Design:**

Semi-structured, individual interviews with five researchers in universities and five employees in the food industry were conducted in the Oslo region, Norway. Interviews were thematically analysed and guided by Braun and Clark.

**Results:**

This study showed positive experiences and attitudes towards a university–industry collaboration within nutrition research aiming for healthier food products. The main facilitators of good collaboration were common goals, the exchange of knowledge and the opportunity for research funding. Barriers to good collaboration were prejudices related to the food industry’s goals and previous experiences of time-consuming projects. Interestingly, collaboration agreements were identified as both facilitators of and barriers to good collaboration.

**Conclusion:**

Stimulating university–food industry collaboration requires increased juridical assistance, provided that the lawyers involved understand the parties’ interests and the need to balance those interests and safeguard mutual trust. In addition, the food industry must take a clearer role in their engagement in public health to improve their trustworthiness in relation to research results.

## Popular scientific summary

- Nutrition research requires a good collaboration between universities and the food industry.
- Industrial partners’ competence in nutrition, health and research was facilitators of good cooperation.
- Collaboration agreements were both facilitators and barriers for good collaboration.
- Stimulating university–food industry collaboration requires increased juridical assistance.

Nutrition research plays an important role in the prevention of non-communicable diseases (1). Product innovations and reformulations increase the availability and purchasing of healthy foods and, thus, contribute to healthier and more sustainable food systems (2, 3). However, nutrition research aiming for more healthy and sustainable food products requires collaboration between university and the food industry (3). Conducting research has traditionally been the role of universities for several decades. However, this role has been challenged by industrial organisations in response to deep changes in the pace and nature of the innovation process (4). Collaboration between universities and industry is nowadays seen as essential for technological progress and economic development (4). However, the impact of industry on universities has been a major topic of discourse in higher education, and researchers found the impact of industry on universities to be negative (5).

Previous studies have investigated the drivers for university–industry collaborations (6–13). Studies on university–industry technology transfer indicate that collaborations may provide academic researchers with access to technical support and specialised expertise and facilities that are essential for their research and development activities (6, 7). In addition, collaborations enable university scientists to achieve a better understanding of the nature of firms’ scientific needs. The types and levels of collaboration between universities and industry seem to depend greatly on firm size and on the sectors in which they operate (10, 14, 15). Even though university–industry collaborations are especially important to stimulate product innovation and reformulation (8), there is limited research on facilitators of and barriers to university–industry collaborations in nutrition research.

The food industry is the largest industry in mainland Norway (15). The Norwegian health authorities and calls for research proposals by Norwegian and European research funding bodies emphasise the need for collaboration between universities and the food industry (4, 16). However, the Norwegian private sector gives little financial support to academic research compared to other European countries (15). Most of the collaborative research projects in nutrition research focus on food technology rather than aspects related to health (1). The way academic institutions and industrial partners approach collaboration their collaboration has been investigated in other scientific areas, such as pharmacy and technology (17–19). There is a lack of research in the international literature on how the collaboration between universities and the food industry aiming for healthier food products is experienced. Investigation of university collaboration with the food industry might be of particular interest, as the food industry has been criticised for influencing the results of research projects in their own favour (20, 21). To gain more knowledge and to stimulate collaboration between universities and the food industry, the aim of this study was to explore the facilitators of and barriers to successful collaboration.

## Materials and methods

### Sampling and participants

Five academic researchers in nutrition and food technology and five employees in the food industry were participated in this study. Participants were recruited from the professional network of the authors. Efforts were made to recruit participants with extensive experience in research collaboration between universities and the food industry. They were asked by e-mail by the second author if they were willing to participate in the study. In total, 30 potential participants were asked to participate. The reason for not being interviewed was either that they did not answer the invitation e-mail or that they did not meet the inclusion criteria. Participants received written information about the main aim of the study prior to the interview. They were also informed that the interviews were part of the second author’s master’s thesis. Recruitment was carried out until we observed replication of response, and no new themes emerged from the interviews (22).

Most of the participants (*n* = 9) were currently involved in a university–food industry collaboration. One participant was not currently involved in a collaboration and had had their last collaboration in 2018. Four out of five participants from the food industry had an educational background in nutritional sciences. All the participants were women. Given the small network of nutritional scientists in Norway, the age and working location of the participants are not presented to secure their anonymity. Table 1 presents the relevant background information about participants’ collaboration experiences.

**Table 1:** Background information of the participants

	*n*	Collaboration projects
Academic researcher in nutrition	3	7–12
Academic researcher in food technology	2	15–25
Employee in the food industry	5	2–30

### Data collection

Interviews followed a semi-structured interview guide (Supplementary file) that was developed by the multi-professional project group. The first and second authors pilot-tested the interview guide amongst peers from the university and the food industry. The pilot-test interviews were included in the analysis as only minor adjustments were made in the interview guide. The following interviews were conducted by the second author alone, who did not have any personal relationship with the participants prior to the study. Interviews lasted from 33 to 49 min and were conducted at the working sites of the participants. Ethical approval for the experimental protocol of this study was obtained by the Norwegian Centre for Data Security (Nr. 363874) and in accordance to the Declaration of Helsinki. Participants gave their written informed consent to participate. We followed the consolidated criteria for reporting qualitative studies (COREQ) (23).

### Analysis

**Fig. 1:**
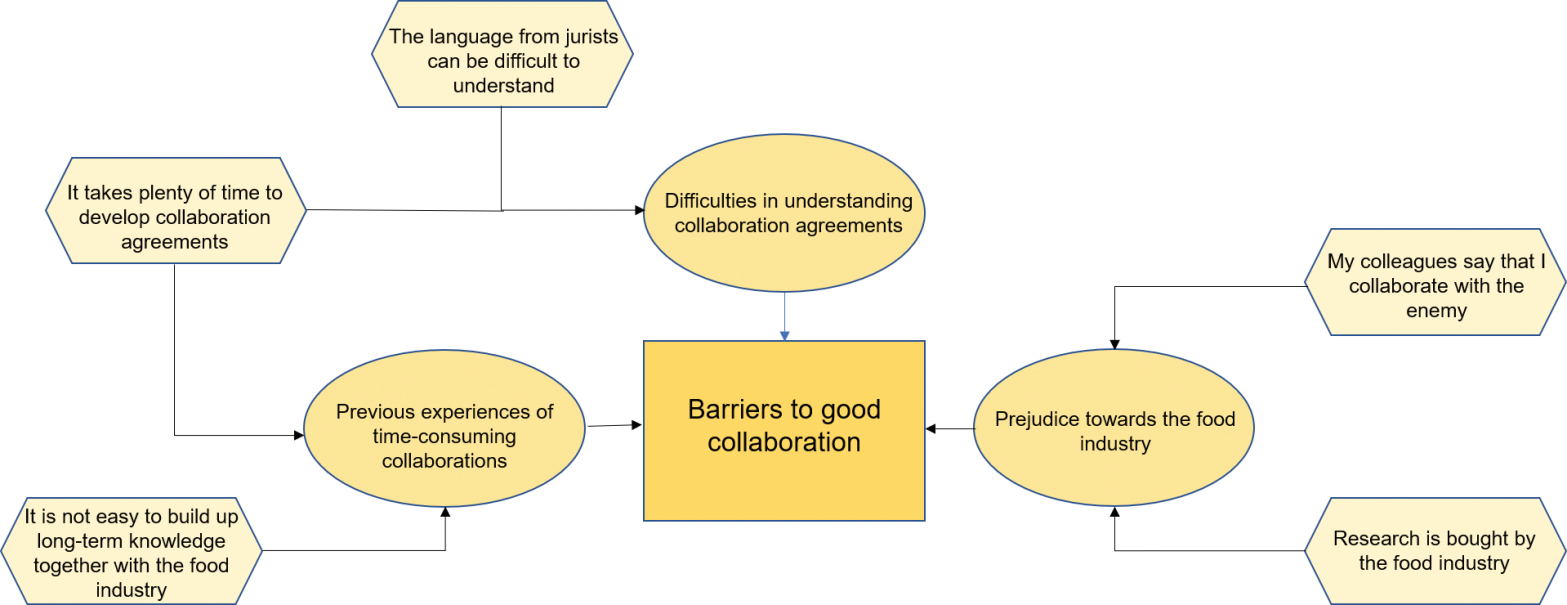
Example of a coding tree.

Interviews were audiotaped and transcribed by the second author. The other authors read the transcripts. The first author randomly compared some of the transcripts with the audiotapes to ensure the accuracy of the transcription process. One participant asked for and read the transcript of his/her interview. The analysis was guided by thematic analysis, according to Braun and Clarke, and included the following steps (22): (1) familiarising themselves with the data by repeated reading of each informant’s transcripts; (2) generating initial codes (words or short phrases in the transcripts) that were relevant for the research questions; (3) organising codes into sub-themes; (4) arranging sub-themes into overarching themes by creating coding trees (see Fig. 1 for an example of a coding tree); and (5) defining and naming the themes. The first, second, and last author conducted the analysis and discussed potential codes and themes with the other authors. A qualitative software program, NVivo (12.0), was used to identify codes and to systematise sub-themes.

## Results

Table 2 summarises the sub-themes and main themes resulting from the data.

**Table 2:** Overview of main themes and sub-themes

Main themes	Sub-themes
Motivation for collaboration	Exchange of knowledge
	Product innovation
	Funding
Facilitators of good collaboration	Common aims and interests in research activities
	Trust
	Competency in research and nutrition amongst industrial partners
	Knowledge of each other
	Collaboration agreements (especially to determine the ownership of results)
Barriers to good collaboration	Difficulties in understanding collaboration agreements
	Previous experiences of time-consuming collaborations
	Prejudice towards the food industry

### Motivation for collaboration

In general, participants from both sides had positive experiences and attitudes towards a university–industry collaboration. Academic researchers and employees from the food industry outlined the exchange of knowledge between the university and the food industry as one of the main motivators for their collaboration. Several participants stated that they had gained a lot of knowledge through previous collaborations, as described by an informant from the food industry:

I experience, or I think that one gains a lot of knowledge from these projects. So, I think that all expectations are fulfilled. (employee food industry)

One participant from the university acknowledged that collaboration with the food industry provided the opportunity to work with real issues:

We get the opportunity to work with real issues; we become closer to the product or what you are actually working with. You often don’t get a good model for what you should work with when you are just working in the lab and not directly with the products or another case. You can practice applicable research when you collaborate with the industry. (academic researcher)

Employees from the food industry also mentioned that their motivation to collaborate with researchers from the university was product innovation:

We have had several research projects on product innovation where we could improve the products for the consumers and that’s very relevant, and we have had research projects where we come up with a new product. We would not have managed that without collaboration. (employee food industry)

For academic researchers, funding of research projects was another motivation for collaboration with the food industry. One academic researcher explained that collaboration with the food industry increases the likelihood of achieving external research funding: ‘Lots of research funding in Norway depends on a collaboration with the industry, either the food industry or another type of industry. So, this is actually necessary for ongoing innovation’. (academic researcher)

### Facilitators of good collaboration

Overall, participants had positive experiences and attitudes towards a university–food industry collaboration. According to our participants, good collaboration presupposed common aims and interests in research projects. In this regard, it appeared to be important that participants experienced their collaboration projects as relevant, as an employee from the food industry stated:

Collaboration is relevant because we are working with topics that are both exciting for researchers and at the same time relevant for the industry. (employee food industry)

Participants from both sides outlined the importance of trust for a good collaboration. However, interviews also revealed that it took time to build trust, exemplified by a statement by an academic researcher with long experience in collaboration:

We work with the same partners because you gain trust and loyalty over time. (academic researcher)

The importance of time and trust was also related to the finding that participants from both sides stated that personal knowledge of each other was important for a good collaboration.

In addition, academic researchers thought that it was important that the collaborators in the food industry had competences in nutrition and research. An academic researcher who had had several collaboration projects with the food industry described it thus:

There should not only be sales people in the industry; you also need people who understand and acknowledge the contribution of research. (academic researcher)

One of the most important facilitators of a good collaboration was a good collaboration agreement between the university and the food industry. One employee from the food industry and three researchers told the interviewer that their leadership required a collaboration agreement prior to the start of the project. Participants from both sides thought that collaboration agreements were especially important in order to determine the ownership of results. Collaboration agreements provided safety to academic researchers by limiting the legal or ethical risks. The participants’ accounts of the contents of these agreements give the impression that the agreements vary in how they regulate the ownership of results. Many participants reported that ownership was held by the academic institution. It should be noted, however, that the participants were not legally trained and could have a mixed understanding of the meaning of ‘ownership’.

### Barriers to good collaboration

Both academic researchers and employees from the food industry experienced collaboration agreements also as barriers to good collaboration. Many participants used templates for collaboration agreements by the Norwegian Research Council or by their institution. However, interviews revealed that the participants from both parts needed juridical support in the establishment of collaboration agreements, as expressed by one academic researcher:

The language from jurists can be difficult to understand for a researcher. So, we depend on jurists from our working sites. (academic researcher)

Participants from both sides felt that overly lengthy and complex collaboration agreements could weaken trust in each other, mainly due to difficulties experienced in understanding the agreements.

Previous experiences of time-consuming collaboration projects were another barrier to good collaboration, and this was mentioned by employees in the food industry. A statement by an academic researcher illustrated that they often had diverse perspectives related to time for a collaboration project:

The problem is that they often think in short terms, while we wish to think more long-term. So, it is not easy to build up long-term knowledge together with the food industry. (academic researcher)

Mistrust in the trustworthiness of research done in collaboration with the food industry was another barrier to good collaboration. Employees in the food industry who had an academic background in nutritional sciences told the interviewer that they had received comments from former research colleagues, such as ‘you have sold your soul’, ‘research is bought by the food industry’ and ‘you collaborate with the enemy’ when they said that they were working in the food industry. These prejudices were also evident in the interviews amongst the academic researchers. Employees in the food industry have found that results from industry-based research were considered less trustworthy without the involvement of the university.

## Discussion

This study showed positive experiences and attitudes towards a university–industry collaboration for nutrition research aiming at developing healthier food products. The main facilitators of good collaboration were common goals, the exchange of knowledge and the opportunity for research funding. Barriers to good collaboration were prejudices related to the food industry’s goals as well as previous experiences of time-consuming projects. Interestingly, collaboration agreements were identified as both facilitators of and barriers to good collaboration.

Universities tend to develop close relations with industry for three main reasons (24). The first is to improve education and research by obtaining a better understanding of process, production, and economic factors. Interestingly, participants from the university in our study did not report improvement in their education as a motivator for collaboration with the industry. The second driver for university–industry collaboration, to diversify sources of financial support, was also mentioned by participants from the university in our study. This finding might be related to the stimulation for university–industry collaboration by national and international health authorities and research funding organisations, such as the European Union (4). By interpreting the results of our study, it has to be acknowledged that our participants relied on research funding by the public and industry sectors. Interviews amongst individuals who conduct nutrition research but do not only rely on financing from other sources may reveal different results. The third is to create new pathways for contributing to the common good. A related facilitator for collaboration amongst our participants from both sides was the exchange of knowledge; however, our participants did not specifically mention the contribution of university–industry collaboration to the development of healthier food systems (5). Bekkers and Freitas identified a variety of channels through which knowledge is being transferred between universities and industry (13). The wide variety of channels through which knowledge between university and industry was transferred was explained by the disciplinary origin and the characteristics of the underlying knowledge. In our study, another important facilitator for good collaboration was that both sides had an educational background in the nutritional sciences. However, this was a contradictory since employees in the food industry had experienced negative attitudes from former research colleagues towards working in the food industry. Our results are in line with the findings of a large-scale survey of UK academic researchers, which found that individual characteristics of researchers have a stronger impact on good collaboration than the characteristics of their departments or universities (11). The focus area of the University department’s research activity was also identified as a major obstacle for university–industry in a qualitative study amongst Italian Academic Departments (12). Interestingly, none of our participants mentioned that their institution had strategic plans to implement the results from research projects.

Our interviews also revealed other barriers to good collaboration. One of the most evident barriers to good collaboration was mistrust in the trustworthiness of results when collaborating with the food industry. In the last decades, the food industry has been criticised for influencing the results of research projects in their own favour (20, 21, 25, 26). However, we have not found any studies on how this mistrust could influence university–food industry collaboration. Zhu et al. outline that a reasonable attitude towards the linkage between university and industry is to seek a balance between conflicting values, for instance regarding the appropriation of research results, instead of isolation (5). A study that investigated conflicts in university–industry collaborations in the field of technology showed that these conflicts often relate to more fundamental challenges, such as the aims of industry and the university, and organisations of diverse people or departments (17). Interestingly, conflicts were not an emerging theme in our analysis. In line with our results, a review article on models and drivers for collaboration between academia and pharmaceutical companies found that understanding and respecting each other’s organisational culture improves the quality of collaboration (18). Another identified barrier was that participants from the food industry mentioned previous experiences of time-consuming collaboration projects. Controversially, in a cross-sectional study, academic authors experienced delay in publication when collaborating with the industry in clinical trials (19). Conducting research has been the traditional role of universities for several decades. Although conducting research takes time, a report from the European Commission emphasises that universities must adjust the pace to the ongoing innovation process (4).

We found that collaboration agreements were both facilitators of and barriers to good university–industry collaboration. This finding may be related to the ‘paradox of formal appropriability mechanism’ uncovered by Miozzo et al. when investigating innovation collaborations of knowledge-intensive business services (27). Miozzo et al.’s study demonstrates the importance of formal, contractual and strategic appropriability in the context of innovation collaboration. In line with our study, agreements that determine the partners’ responsibility facilitated trust and knowledge transfer between the two parties. However, too strict rules by legal departments can limit incentives to the exchange and transfer of knowledge and may be associated with less willingness to undertake collaboration or act as a barrier to knowledge creation and transfer (27). It must be acknowledged that all the participants in our study had difficulties in understanding the agreements. According to the European Commission, the changing role of universities in conducting research with industry requires modernisation of their managerial and organisational skills (4). Results from our study indicate that researchers in universities need more juridical assistance to facilitate more collaboration with the food industry. The lawyers drafting the collaboration agreements should understand the interests of both parties, be aware that mutual trust is important for the projects to succeed and that rigid, complicated and unbalanced agreements may pose a threat to that trust. Even though our participants had not experienced collaboration conflicts related to funding, Van Wee argues for the need for a code of conduct for researchers and their funders to secure indirect pressure from funders (28). This might be particularly important when collaborating with the food industry that has been criticised for influencing the results of research projects in their own favour (20, 21).

### Study limitations

This study was conducted with a small sample size, which is typical of qualitative studies (29). To secure privacy and due to the small environment in nutritional science, we cannot provide more background information of the participants. It must be acknowledged that the researchers’ professional backgrounds and personal experiences might have shaped the gathering and interpretation of data. However, we have involved researchers with experience from the food industry and research and development contracts to limit the possible bias of a single researcher’s preconceptions on the data collection and interpretation of the results. The interviews were conducted by the second author, who was a master’s student in Public Health Nutrition without previous experience in qualitative research. She was closely supervised by the first author, who has a PhD and extensive experience in individual interviewing. Even though the recruitment was carried out until we observed replication in the responses (22), interviews with participants with other experiences of university–food industry collaboration might have revealed additional themes. Nonetheless, we believe that our findings may be transferable to university–food industry collaborations in a similar context to the Norwegian food system.

## Conclusions

This study demonstrated positive attitudes and motivation towards a university–food industry collaboration. Facilitators of good cooperation were easily understand collaboration agreements and mutual trust, and the industrial partners have competence in nutrition, health and/or research. Stimulating university–food industry collaboration requires increased juridical assistance, provided that the lawyers were involved understanding the parties’ interests and the need to balance those interests and safeguard mutual trust. In addition, the food industry must take a clearer role in their engagement in public health to improve their trustworthiness in relation to research results.

## Credit author statement

LGH (PhD), HSL (MSc), HIJ (PhD) and VHTH (PhD) designed the research. LGH and HSL conducted and analysed the interviews. LGH, HSL, HIJ and VHTH contributed to the interpretation of the data. LGH drafted the manuscript. LGH, HIJ and VHTH had primary responsibility for the final content. All authors read and approved the final manuscript.
